# Frailty and Nutritional Status among Urban Older Adults in South India

**DOI:** 10.1155/2020/8763413

**Published:** 2020-07-10

**Authors:** T. Shalini, P. Swathi Chitra, B. Naveen Kumar, G. Madhavi, G. Bhanuprakash Reddy

**Affiliations:** ^1^Department of Biochemistry, ICMR-National Institute of Nutrition, Jamai-Osmania, Tarnaka, Hyderabad, Telangana, India; ^2^Department of Statistics, ICMR-National Institute of Nutrition, Jamai-Osmania, Tarnaka, Hyderabad, Telangana, India; ^3^Department of Community Studies, ICMR-National Institute of Nutrition, Jamai-Osmania, Tarnaka, Hyderabad, Telangana, India

## Abstract

The purpose of this study was to assess the prevalence of frailty and nutritional status among older adults. This population-based cross-sectional study was conducted in 163 subjects aged 60–88 years, from Hyderabad City, South India. Data were obtained on sociodemographic details and anthropometry and biochemical parameters. Dietary intake was assessed by a three-day 24 h dietary recall, and the probability of adequacy (PA) was calculated using the estimated average requirements. Frailty indicators were as follows: handgrip strength was measured by using a Jamar dynamometer, gait speed was measured by a ten-meter length walk test, and low physical activity level, weight loss, and exhaustion were assessed using a questionnaire. Among the study population, 20% of the participants were frail and 80% were nonfrail. The prevalence of frailty is higher in older (30.1%) than the younger (12.2%) age groups, and it is more so in women (32.4%) than in men (10.1%). The lower educational status and income were associated with frailty. The PA of most of the nutrients was low in the frail group. Noticeably, the mean PA (MPA) across the fourteen micronutrients was significantly higher in nonfrail (38%) compared to the frail group (25%). The prevalence of frailty was higher in the lowest tertile of most of the food groups and nutrient intake compared to the highest tertile. The study revealed a 20% prevalence of frailty among urban older adults and provided evidence that inadequate intake of nutrients is independently associated with frailty.

## 1. Introduction 

The average life span of humans at birth has been increased in the last century, approximately from 45 years (the early 1900s) to 80 years today. It is estimated that by 2050, about 21.5% (∼2 billion) of the global population will be over 60 years of age [1, 2]. This demographic transition of increased life expectancy is associated with the burden of several age-related disorders, including frailty [3]. Frailty, a biologic or geriatric syndrome characterized by multisystem dysregulation leading to a loss of dynamic homeostasis, decreased physiological, functional, and cognitive reserves that confer vulnerability to adverse outcomes. Frail people are at a higher risk of disability, falls, cognitive impairment, hospitalization, dependence, and mortality, hence becoming a major clinical and public health concern [3, 4].

There are over 25 subjective and objective frailty assessment methods developed globally to assess frailty with many different intangible definitions. The most followed methods of measurement include the Rockwood frailty index, which defines frailty as a result of several age-related deficits that may lead to poor health [5]. The second method developed by Fried defines frailty as a unidimensional, principally physical domain which includes three of five indicators such as exhaustion, weak grip strength, low energy expenditure, slow walking speed, and weight loss [6]. However, other researchers have proposed to include the cognitive domain, a multidimensional construct, which could aid in a better understanding of the frailty phenotypes and pathways to adverse outcomes [7].

The quality of life of aging people can be improved if intervened at an early stage of functional decline by slowing, delaying, or partly reversing the state of frailty, if assessed appropriately [8]. Poor nutritional status is one of the factors found to be associated with frailty, which might be due to insufficient food intake. Epidemiological studies have reported that dietary protein content, protein quality, and micronutrients could play a crucial role in the development and management of aging and frailty [9]. Moreover, adequate intakes of macronutrients and micronutrients have been found to reduce the risk of frailty [10].

In developing countries such as India, frailty assessment among older adults has seldom received the attention of the investigators. Few studies reported varied prevalence (16.3–55.5%) of frailty in India [11–13], while some studies emphasized on physical, cognitive, and depression domains separately [14, 15], but a comprehensive approach towards frailty assessment, particularly nutritional component, is missing. Therefore, the present study was conducted (i) to assess the prevalence of frailty among urban older adults using the Fried frailty phenotype criteria and (ii) to assess their nutritional status.

## 2. Methods

### 2.1. Study Design, Sample Size, and Recruitment of Subjects

This population-based cross-sectional study was conducted among older adults aged 60 years and above in the urban areas of Hyderabad Metro City, Telangana State, India, from November 2016 to July 2017. Based on the reported prevalence of frailty among older adults as 56%, the sample size was calculated [11]. Assuming a 95% confidence interval (CI) with a relative precision of 20%, the sample size arrived was 78. However, with a design effect of 2, the sample size comes to 156.

**Figure 1:**
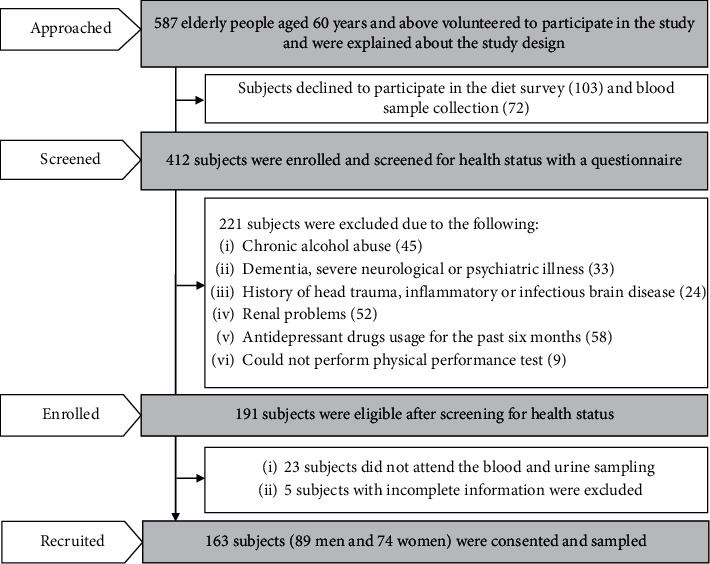
Flowchart showing the recruitment and selection of study participants.

The Hyderabad City was stratified into four zones (south, east, west, and north), and two wards were selected from each zone by a simple random sampling procedure to capture the entire population of the city. To enroll participants, health camps were organized at randomly selected wards. From each ward, four locations were selected, and in each location, one health camp was organized. Approximately, 20 subjects were approached in each health camp. The details of the selection and recruitment of the study participants are depicted in Figure 1. A total of 163 participants, 89 men and 74 women, who fulfilled the criteria (mentioned in Figure 1) have consented for participation.

The study was conducted according to the guidelines laid down in the 1964 Declaration of Helsinki and its later amendments. All procedures involving human participants were approved by the Institutional Ethics Committee (ethical approval number: IEC; # CR9/I/2014). Written informed consent (or thumb impression in the case of illiterates) was obtained from the participants who volunteered to participate in the study.

### 2.2. Data Collection

Sociodemographic information such as age, literacy status, cohabitate details, food habits, and self-reported comorbid conditions was obtained using a questionnaire. Participants were categorized into two groups based on their food habits. Those who never consumed animal foods (such as poultry, meat, eggs, and fish) were included in the vegetarian group, and the others who consumed both animal foods and plant foods were included in the mixed diet group.

#### 2.2.1. Anthropometric Measurements

The body weight and height were recorded using the SECA weighing scale and anthropometric rod, respectively, and the body mass index (BMI) was calculated. Waist circumference (WC) was measured using a fiber-reinforced nonelastic tape at a point midway between the lower rib region and the iliac crest, and hip circumference (HC) was measured by passing the tape over maximum protuberance on buttocks. Asian cutoff values were used for BMI classification [16] and defining abdominal and central obesity [17]. Blood pressure (BP) was measured thrice with a five-minute interval between each measurement using a BP apparatus, and the average of three readings was taken. Participants with systolic blood pressure (SBP) of ≥140 mmHg and diastolic blood pressure (DBP) of ≥90 mmHg and/or those participants on antihypertensive medication were considered hypertensive [18].

#### 2.2.2. Biochemical Estimations

Fasting venous blood samples were collected in heparin tubes early in the morning following overnight fast, and spot urine samples (first-morning void) were collected in sterile urine containers. The samples were transported to the laboratory in the icebox for further analysis. Blood and plasma were separated by centrifugation at 3500 rpm for 10 mins. Fasting blood glucose (FBG) was estimated in whole blood using an Accu-Chek Active glucometer [19]. Glycosylated haemoglobin (HbA1c) was estimated by an Afinion AS100 Analyzer (Axis-Shield, Norway) based on the principle of boronate affinity [20] and haemoglobin (Hb) by the cyanmethaemoglobin method using a spectrophotometer (Shimadzu UV 2600). Lipid profile (high-density lipoprotein (HDL), total cholesterol (TC), and triglycerides (TG)) was analyzed in plasma using commercially available kits from BioSystems (Barcelona, Spain). Low-density lipoprotein (LDL) concentrations were calculated using the Friedewald formula [21]. Urinary albumin was quantified using a solid-phase immunochemical assay and urinary creatinine by an enzymatic colourimetric test in a fully automated Afinion AS100 Analyzer (Axis-Shield, Norway) [22], and then the urinary albumin-to-creatinine ratio (UACR) (expressed as mg/g creatinine) was calculated.

#### 2.2.3. Cutoffs for Covariates

FBG < 110 mg/dL was considered as normal, 110–125 mg/dL as impaired fasting glucose (IFG), and ≥126 mg/dL as diabetic [23]. An HbA1c value of < 6.5% was considered as normal [24]. The prevalence of anemia was calculated based on the Hb levels. Anemia was defined as a Hb value < 13.0 g/dL in men and <12.0 g/dL in women [25]. The optimal plasma concentration of TC was <200 mg/dL, <130 mg/dL for LDL, <150 mg/dL for TG, and low HDL was ≥40 mg/dL in men and ≥50 mg/dL in women [26]. According to the National Kidney Foundation, UACR < 30 mg/g was considered as normal, 30–300 mg/g as microalbuminuria, and ≥300 mg/g as overt nephropathy [27].

#### 2.2.4. Dietary Assessment

Individual dietary intake was assessed in a subset of the samples (*n* = 88, 48 men and 40 women) using a systematic random sampling procedure. A 24 h dietary recall was carried out on three different days (2 nonconsecutive weekdays and one weekend day) to capture intra- and interindividual variation [28]. The member of the household who cooked food for the entire household was interviewed for dietary intake of individuals during the previous 24 h, excluding festival, function, and fasting days. A standardized set of twelve cups and two spoons were used as visual tools for assessing portion sizes [29]. The raw ingredients used for the food preparation were weighed using a portable electronic digital diet scale (Seca Culina 852®). The quantities of raw foods were computed from the intakes of cooked foods (intakes of the raw food by the individual = (quantity of raw food in the preparation/total cooked quantity of food) × individual intake of cooked food).

The nutritive value of raw foods was calculated using the Indian Food-Composition Tables (IFCTs) [30], while the United States Department of Agriculture Food and Nutrient Database [31] was used for those foods that did not have a nutrient value in the IFCT. After correction for moisture, the nutritive values of these two databases were comparable (within 10–20% variation). The total daily consumption was computed based on the above mentioned nutritive value databases and by taking the average of 3 days of diet survey, using the in-house software.

*(1). Probability of Adequacy (PA)*. The adequacy of micronutrients was assessed using the probability approach which relates an individual's usual intake of nutrients to the distribution of requirements for a particular life stage and gender group using estimated average requirement (EAR) values and its standard deviation (SD) [32–34]. Then, the PA was computed using the “CDFNORM” function in SPSS software. CDFNORM function is a cumulative probability distribution of nutrient requirements, assumed to be a normal distribution, and is expressed as area under the probability curve. This function computes by plotting each individual's intake data from the study population and constructs a risk curve using the requirement (EAR and SD) distribution of the group (*Z* score = (intake − EAR)/SD of the requirement). Then, the risk curve was compared to the distribution of intakes of the study population to determine what proportion of the population has an inadequate intake. Thus, PA determines the probability that an individual's intake in a group meets the requirements, and then, their mean of the individual probabilities is obtained, which is used to estimate the prevalence of adequacy of a particular nutrient [34, 35]. Hence, the micronutrient adequacy was evaluated by calculating the PA for fourteen micronutrients that are of public health importance in this study: vitamins such as A, C, B1, B2, niacin, B6, folate, and B12, and minerals such as calcium, zinc, iron, magnesium, phosphorus, and selenium. The recommended EAR, as set by the Institute of Medicine (IOM) (National Academies, Food and Nutrition Board) [36], according to the sex and age group, was considered for the calculation of PA. The resulting value for PA ranged from 0 to 100%, and an overall mean PA (MPA) was calculated by averaging the PA across the fourteen nutrients. The prevalence of inadequacy was defined by considering MPA below 50% (MPA < 0.5) [36, 37].

(2) *Nutrient Density*. Nutrient density is the ratio of the amount of nutrient intake in the diet to the energy provided by the same diet and is expressed as the amount of the nutrient per 1,000 kcal of energy [38].

### 2.3. Frailty Indicators

Five indicators were assessed to measure frailty. These included (i) weakness, (ii) weight loss, (iii) physical activity level, (iv) exhaustion, and (v) gait speed (GS).

#### 2.3.1. Weakness

Handgrip strength (HGS) was measured to estimate the physical weakness of the participant using a Jamar hand-held dynamometer [6]. The cutoff for HGS stratified by gender and BMI is depicted in Supplementary Table 1A. To determine the cutoff point for defining the lowest quartile on measures of HGS, the 25^th^ percentile was used [39].

#### 2.3.2. Weight Loss

Self-reported unintentional weight loss was assessed in response to the question, “Have you lost any weight during the past 12 months?” Those reporting a weight loss of 4.5 kg or more in the previous year were considered [6].

#### 2.3.3. Low Physical Activity Level

A question was asked to the participants, “Taking into account both work and leisure, would you say that you are very, fairly, not very, or not at all physically active?” Those reported themselves as not very or not at all physically active were considered physically inactive [7].

#### 2.3.4. Exhaustion

A question was asked to the participants, “Are you feeling worn out or exhausted?” The participants who reported themselves as exhausted were considered as exhausted [7].

#### 2.3.5. Gait Speed (GS)

It was assessed by a standard timed walking test in which a five-meter length of the string was laid along the ground, and the participants were asked to get up from the chair and walk normally to the end of the string, turn round and walk back again, and sit on the chair [7]. Due to the change in stride length of a person, the GS varies with height. The cutoff for GS stratified by gender and height is depicted in Supplementary Table 1B. To determine the cutoff point for defining the lowest quartile on measures of GS, the 25^th^ percentile was used [39].

On a scale of 5, a person who gets a score of 0–2 was categorized as nonfrail and 3–5 score as a frail person [6, 40].

### 2.4. Statistical Analyses

Data analyses were performed using the SPSS software package (version 19.0, SPSS Inc, Chicago, IL). As most of the data were skewed, the anthropometric parameters, clinical variables, food groups, and nutrients by frailty status were reported using medians and 25^th^(*P*_25_) and 75^th^(*P*_75_) percentiles, and comparisons for the same were carried out by the Mann–Whitney *U* test. The median values of the variables (HGS and GS) were compared across the age groups and gender using a Kruskal–Wallis test with pairwise-multiple comparisons. The chi-square (*χ*^2^) test was used for testing the association between categorical variables. Student's *t*-test was used to compare the PA and MPA by frailty status. The food groups and nutrients were divided into tertiles, and the associations between frailty status and the dietary variables were examined using the *χ*^2^ test. Statistical significance was considered at *P* < 0.05.

## 3. Results

The median (*P*_25_–*P*_75_) age of the participants was 65.0 (62.0–70.0) (Table 1). The gender (men, 55%; women, 45%) and the age-wise (60–65 years, 55%; ≥66 years, 45%) distributions were almost similar in both groups. The majority of the subjects were baccalaureate graduates and above (35.2%) and were consuming mixed diets (72%). About 3% of the subjects were underweight, 24% had a normal BMI, and 73% were overweight and obese.

### 3.1. Prevalence of Frailty and Its Association with Age and Gender

**Figure 2:**
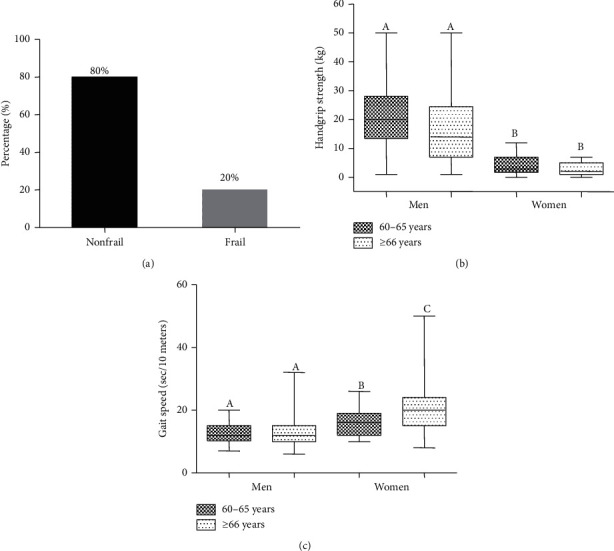
Prevalence of frailty (a), the relation of handgrip strength (b), and gait speed (c) with age and gender. Significant differences (*P* < 0.05) of median values with age and gender are indicated by different superscript letters (A, B, and C) above the bars.

According to Fried frailty phenotype criteria, 20% of the study participants were frail and 80% were nonfrail (Figure 2(a)). We determined the association of two direct measures of frailty (HGS and GS) with age and gender. The HGS was observed to be lower with increasing age considering both genders, though it was not significant. The HGS was significantly higher in men compared to women in both the age groups (Figure 2(b)). There was no difference in GS between the age groups among men, but there was a difference between the age groups among women. The GS of men was significantly different when compared to women of respective age groups (Figure 2(c)).

### 3.2. Nutritional Status of the Study Participants by Frailty Status

Median age and UACR were significantly higher, whereas Hb was significantly lower in the frail group compared to the nonfrail group. No significant (*P* < 0.05) difference was observed for other variables (Table 1).

The prevalence of frailty in the ≥66-year age group (30.1%) was significantly higher when compared to the 60–65-year age group (12.2%). By gender, the prevalence of frailty was significantly higher in women (32.4%) than men (10.1%). Nonearning (41.5%) participants had a significantly higher prevalence compared to earning (6.4%) participants. Uneducated and participants of lower education had a significantly higher prevalence of frailty compared to those who had higher education (Table 2).

The median (*P*_25_–*P*_75_) intakes of cereals and millets, pulses and legumes, green leafy vegetables, roots and tubers, nuts and oilseeds, spices and condiments, fruits, and fats and oils and all the nutrients except for vitamin B12 were significantly lower in the frail group compared to the nonfrail group (Table 3). Dietary energy intake, a proxy for food intake, was significantly low in the frail group. The major contributor to energy was carbohydrates (∼56%) and fat (∼28%), and the protein intake was near to optimal (∼11%) in both the groups (Table 3).

### 3.3. Probability of Adequacy by Frailty Status

**Figure 3:**
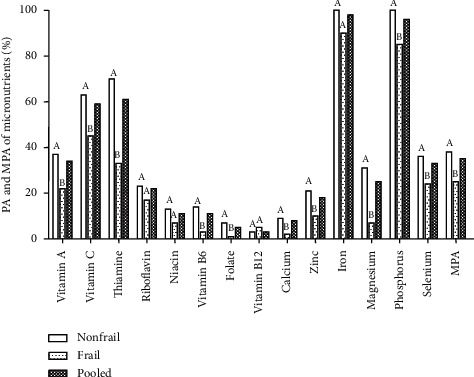
Probability of adequacy and mean probability of adequacy of micronutrients among nonfrail and frail participants. PA, probability of adequacy; MPA, mean probability of adequacy. Pooled data represent the total number of samples (*n* = 88). Mean values between the groups were compared by Student's *t*-test. Data represent (%) adequacy, and significant differences (*P* < 0.05) of mean values between the groups are indicated by different superscript letters (A and B) above the bars.

**Figure 4:**
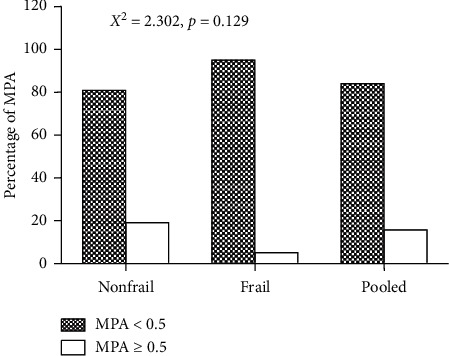
Association of the mean probability of adequacy (MPA) with frailty status. Data represent % inadequacy (<0.5) and % adequacy (≥0.5) of micronutrients. Pooled data represent the total number of samples (*n* = 88). *P* < 0.05 was considered to be significant.

The PA of vitamin A (*P*=0.038), vitamin C (*P*=0.040), thiamine (*P*=0.001), folate (*P*=0.013), vitamin B6 (*P*=0.003), calcium (*P*=0.013), zinc (*P*=0.042), magnesium (*P* < 0.001), phosphorus (*P*=0.01), and selenium (*P*=0.042) was significantly lower in the frail group compared to the nonfrail group (Figure 3). Noticeably, the MPA across the fourteen micronutrients was 35% and was significantly higher in nonfrail (38%) compared to the frail group (25%) (Figure 3). The risk of micronutrient inadequacy (MPA < 0.5) was about 84% in the study subjects and was associated with frailty status but not significant (*P* < 0.05) (Figure 4). The prevalence of inadequacy (MPA < 0.5) was higher in the frail group (95%) compared to the nonfrail group (81%) (Figure 4).

### 3.4. Food and Nutrient Quality of the Participants by Frailty Status

The nutrient density and nutrients per kg body weight were similar between frail and nonfrail groups (Supplementary Table 2).

### 3.5. Association of Food Groups and Nutrient Intake with Frailty

Association of food groups and nutrient intake according to the tertiles in frail participants is shown in Supplementary Tables 3A and 3B. Significantly high prevalence of frailty was observed in the lowest tertile intakes of most of the food groups and nutrients compared to the highest tertile. Model 1 (adjusted for age and gender) and model 2 (adjusted for age, gender, and energy) adjustments did not result in any change in the existing associations.

## 4. Discussion

Proper nutrition plays an essential role in maintaining good health. As nutritional status is an essential factor contributing to frailty, inadequate food intake among older adults, due to dentition problems, anorexia nervosa, social isolation, and economic hardships, makes them predisposed to frailty. If assessed beforehand, the process of frailty among older adults can be postponed, and hence, they may be provided with a healthy life.

Studies reported in India showed a varied prevalence (16.3–55.5%) of frailty [11–13]. In the present study, the prevalence of frailty was 20%. This low-end prevalence of frailty in this study may be due to age, ethnicity, and dietary habits compared to the reported studies that were done in higher age groups (65 years).

Physical inactivity results in loss of muscle mass due to the imbalance between synthesis and degradation of muscle proteins, even in healthy older adults. This situation can worsen owing to the steady loss of metabolic reserves and functional capacity. Handgrip strength and GS were found to be the predictors of physical functional status and all-cause mortality in older adults [41]. This study has shown a trend in HGS reduction with increasing age, and men were stronger than women, in concurrence with the previous studies [39]. This might be due to a decrease in the number and size of muscle fibers with progressing age (type II) and the difference in dietary patterns (especially protein intake). A study reported that older adults in India had significantly poor muscle strength than in the United States [14]. A cross-sectional study among older North Indians revealed that the GS was found to be lower with increasing age and higher with increasing height in both genders [39]. In agreement with this report, we have observed similar findings in the present study.

The findings of the current study concur with the recent studies that the prevalence of frailty increased with age, more so in women and participants having lower education and income [6, 11]. A Study on global AGEing and adult health (SAGE) revealed that in low- and middle-countries, both frailty and disability increased with age and were higher in women [11]. In a Cardiovascular Health Study (CHS) and SAGE study, education and income were found to be related to frailty [11, 42]. The gender difference in frailty may be due to lower lean mass, strength, differences in patterns of physical activity and performance, longer life expectancy, and higher morbidity rates and more likely to live alone with the consequence of poor nutrition in women than men.

The adequacy of most of the nutrients was low in the frail group, and the MPA across the fourteen micronutrients was 25%. The prevalence of frailty was observed to be higher in the lowest tertile of most of the nutrient intake and was almost double compared to the highest tertile. The associations remained the same even after adjusted for model 1 (age and gender) and model 2 (age, gender, and energy). Similarly, Health, Aging, and Body Composition (Health-ABC) study among community-dwelling older adults found that low intake of dietary protein was associated with a 40% loss of lean body mass [43] and the protein intake is the primary factor responsible for muscle protein anabolism. Low intake of protein and vitamins D, E, C, and folate after adjusted for energy intake has been shown to be independently associated with frailty in the InCHIANTI study [10]. Impairment of mitochondrial function is a hallmark of frailty development [44], which may be influenced by the deficiency of micronutrients. The depletion of mitochondrial function in muscles causes diminished energy production, which may lead to fatigue and weakness in frail individuals. Likewise, in the present study, inadequate intakes by the frail subjects as apparent by a higher prevalence of inadequacy (MPA < 0.5) (95%) might affect the mitochondrial function in muscles which are in turn responsible for the increased physical inactivity in frail subjects. Though the amount of food is low in the frail group, the quality of diet is almost equal in both groups as evidenced by nutrient density.

The consumption of fruits and vegetables (rich in micronutrients, antioxidants, and fiber) was observed to be low in frail older adults. A study demonstrated that the consumption of three portions of fruits and two portions of vegetables per day was related to a lower risk of frailty [45]. Another study reported an association between antioxidant deficiency and reduced muscle strength [10]. A similar relation was observed in the present study, wherein lower intakes of antioxidant vitamins (A and C) and minerals (zinc and selenium) were associated with frailty.

In conclusion, 20% of the study population was frail, the risk of frailty increased with increasing age, and the women are predisposed more than men. The significant determinants associated with frailty were lower educational status and income. Dietary intakes of food groups and the majority of nutrients were found to be low in frail participants. The prevalence of inadequacy (MPA < 0.5) was about 95% in the frail group. The findings of the study demonstrated that inadequate nutritional intake could be a contributing factor to frailty among older adults.

## 5. Strengths and Limitations

This is the first study in India that reports the prevalence of frailty and its association with nutritional status among the urban older adults in South India. These findings contribute to the current knowledge of the prevalence of frailty and understanding its association with nutritional status. The diet calculations used in the study do not account for cooking losses. The results are based on the raw data analysis and are independent of sampling weights and are not adjusted for inflated SDs resulting from complex sampling design. The present study population might not be a representation of the entire country concerning geography, food habits, and other cultural variations, which highlights the need for further studies with larger cohorts to substantiate these findings.

## Figures and Tables

**Table 1:** Comparison of anthropometric details and blood parameters between nonfrail and frail participants.

Parameter	Pooled (*n* = 163), median (*P*_25_–*P*_75_)	Nonfrail (*n* = 130), median (*P*_25_–*P*_75_)	Frail (*n* = 33), median (*P*_25_–*P*_75_)	*P* value
Age, years	65.0 (62.0–70.0)	64.0 (61.0–68.0)	70.0 (65.0–76.0)	<0.001
Height, cm	160 (152–166)	161 (154–167)	155 (150–163)	0.032
Weight, kg	66.6 (58.6–73.7)	66.9 (59.2–73.7)	61.1 (49.0–72.3)	0.078
BMI, kg/m^2^	25.7 (22.7–28.5)	26.0 (23.0–28.5)	24.3 (21.7–29.2)	0.243
WC, cm	94.0 (85.1–101.6)	94.0 (86.4–101.6)	91.4 (83.8–99.1)	0.329
HC, cm	99.1 (92.2–106.7)	99.1 (94.0–106.7)	98.3 (90.2–106.7)	0.709
WHR	0.95 (0.89–0.99)	0.95 (0.9–1.0)	0.93 (0.87–0.98)	0.214
SBP, mmHg	140.5 (130.0–162.0)	140.0 (128.0–160.0)	150.0 (134.0–171.0)	0.079
DBP, mmHg	83.0 (76.0–91.0)	81.0 (76.0–90.0)	85.0 (77.0–92.0)	0.634
FBG, mg/dl	110.0 (97.0–136.0)	108.5 (98.0–135.0)	112 (97.0–147.0)	0.705
TC, mg/dl	176.7 (144.2–208.0)	177.8 (148.2–209.6)	171.3 (139.3–198.8)	0.449
HDL, mg/dl	41.7 (33.4–49.3)	41.0 (33.0–48.9)	45.8 (34.7–50.3)	0.273
LDL, mg/dl	110.4 (83.1–141.0)	111.3 (84.0–145.5)	110.1 (76.0–128.7)	0.327
TG, mg/dl	101.7 (76.7–141.5)	103.4 (78.7–144.0)	96.1 (69.9–129.7)	0.451
Hb, g/dl	13.4 (12.4–14.6)	13.6 (12.6–14.7)	12.8 (11.9–13.6)	0.011
HbA1c, (%)	6.4 (5.8–7.4)	6.4 (5.8–7.4)	6.5 (6.0–7.7)	0.202
Creatinine, mg/dl	1.0 (0.9–1.1)	1.0 (0.9–1.1)	1.0 (0.9–1.1)	0.826
UACR, mg/g creatinine	15.1 (8.3–35.2)	13.4 (8.1–29.8)	24.6 (13.6–88.9)	0.009

BMI: body mass index; WC: waist circumference; WHR: waist-to-hip ratio; SBP: systolic blood pressure; DBP: diastolic blood pressure; FBG: fasting blood glucose; TC: total cholesterol; HDL: high-density lipoprotein cholesterol; LDL: low-density lipoprotein cholesterol; TG: triglycerides; Hb: haemoglobin; HbA1c: glycosylated haemoglobin; UACR: urinary albumin-to-creatinine ratio; *P*_25_: 25^th^ percentile; *P*_75_: 75^th^ percentile. Values represent medians, 25^th^ and 75^th^ percentiles. *P* < 0.05 was considered to be significant.

**Table 2:** Association of sociodemographic details and anthropometry and blood parameters with frailty status.

Parameter	Pooled (*n* = 163)	Nonfrail (*n* = 130)	Frail (*n* = 33)	*P* value
Age, years				
60–65	90	79 (87.8%)	11 (12.2%)	0.005
≥66	73	51 (69.9%)	22 (30.1%)	
Gender				
Men	89	80 (89.9%)	9 (10.1%)	<0.001
Women	74	50 (67.6%)	24 (32.4%)	
Food habits				
Vegetarian	45	32 (71.1%)	13 (28.8%)	0.426
Mixed diet	118	91 (77.1%)	27 (22.9%)	
Occupation				
Earning	47	44 (93.6%)	3 (6.4%)	<0.001
Nonearning	41	24 (58.5%)	17 (41.5%)	
Education				
Uneducated	7	4 (57.1%)	3 (42.9%)	<0.001
1–8 standard	20	9 (45.0%)	11 (55.0%)	
9–12 standard	30	26 (86.7%)	4 (13.3%)	
Graduation and above	31	29 (93.5%)	2 (6.5%)	
BMI, kg/m^2^				
<18.5	5	3 (60.0%)	2 (40.0%)	0.300
18.5–23	39	29 (74.4%)	10 (25.6%)	
≥23	119	98 (82.4%)	21 (17.6%)	
WC, cm				
Normal	29	20 (69.0%)	9 (31.0%)	0.279
Abdominal obesity	94	74 (78.7%)	20 (21.3%)	
WHR				
Normal	6	3 (50.0%)	3 (50.0%)	0.118
Central obesity	117	91 (77.8%)	26 (22.2%)	
Hypertension (HTN), mmHg				
Normal (SBP <140, DBP <90)	114	93 (81.6%)	21 (18.4%)	0.377
HTN (SBP ≥140, DBP ≥90)	49	37 (75.5%)	12 (24.5%)	
FBG, mg/dl				
<110	83	68 (81.9%)	15 (18.1%)	0.619
110–125	32	26 (81.3%)	6 (18.8%)	
≥126	48	36 (75.0%)	12 (25.0%)	
TC, mg/dl				
<200	113	88 (77.9%)	25 (22.1%)	0.370
≥200	50	42 (84.0%)	8 (16.0%)	
TG, mg/dl				
<150	130	102 (78.5%)	28 (21.5%)	0.415
≥150	33	28 (84.8%)	5 (15.2%)	
HDL, mg/dl				
Male: <40; female: <50	97	77 (79.4%)	20 (20.6%)	0.886
Male: ≥40; female: ≥50	66	53 (80.3%)	13 (19.7%)	
LDL, mg/dl				
<130	131	102 (77.9%)	29 (22.1%)	0.224
≥130	32	28 (87.5%)	4 (12.5%)	
Hb, g/dl				
Male: <13; female: <12	30	23 (76.7%)	7 (23.3%)	0.497
Male: ≥13; female: ≥12	113	93 (82.3%)	20 (17.7%)	
HbA1c (%)				
<6.5	94	74 (78.7%)	20 (21.3%)	0.295
≥6.5	69	55 (79.7%)	14 (20.3%)	
UACR, mg/g				
<30	77	63 (81.8%)	14 (18.2%)	0.097
30–300	29	18 (62.1%)	11 (37.9%)	
≥300	3	2 (66.7%)	1 (33.3%)	
Diabetes				
Yes	62	46 (74.2%)	16 (25.8%)	0.639
No	63	49 (77.8%)	14 (22.2%)	
Cataract				
Yes	37	26 (70.3%)	11 (29.7%)	0.277
No	87	69 (79.3%)	18 (20.7%)	
Osteoarthritis				
Yes	36	24 (66.7%)	12 (33.3%)	0.094
No	88	71 (80.7%)	17 (19.3%)	

BMI: body mass index; WC: waist circumference; WHR: waist-to-hip ratio; HTN: hypertension; SBP: systolic blood pressure; DBP: diastolic blood pressure; FBG: fasting blood glucose; TC: total cholesterol; HDL: high-density lipoprotein cholesterol; LDL: low-density lipoprotein cholesterol; TG: triglycerides; Hb: haemoglobin; HbA1c: glycosylated haemoglobin; UACR: urinary albumin-to-creatinine ratio. Values represent percentages (%). *P* < 0.05 was considered to be significant.

**Table 3:** Median (*P*_25_–*P*_75_) intake of food groups and nutrients by frailty status.

Food groups/nutrients	Pooled (*n* = 88), median (*P*_25_–*P*_75_)	Nonfrail (*n* = 68), median (*P*_25_–*P*_75_)	Frail (*n* = 20), median (*P*_25_–*P*_75_)	*P* value
*Food groups*				
Cereals and millets (g)	231.4 (205.2–277.9)	250.2 (208.7–289.6)	204.8 (188.0–227.9)	0.001
Pulses and legumes (g)	40.1 (25.4–47.5)	42.3 (32.2–51.5)	24.8 (17.9–36.5)	<0.001
Green leafy vegetables (g)	17.1 (11.4–30.9)	18.0 (13.5–34.2)	11.9 (6.8–23.4)	0.014
Other vegetables (g)	130.9 (78.7–176.2)	131.8 (83.8–177.2)	110.9 (62.9–173.2)	0.504
Roots and tubers (g)	56.5 (42.4–79.7)	59.4 (46.1–84.1)	47.1 (33.7–60.1)	0.037
Nuts and oilseeds (g)	8.7 (4.6–13.6)	9.9 (5.2–14.2)	5.2 (3.8–8.2)	0.007
Spices and condiments (g)	10.4 (8.5–12.8)	11.3 (9.4–13.2)	7.7 (5.1–10.2)	<0.001
Fruits (g)	113.2 (84.4–189.9)	124.5 (92.2–198.6)	100.8 (63.2–135.8)	0.019
Animal foods (g)	20.8 (0.0–52.6)	24.0 (0.0–58.5)	20.8 (0.0–52.6)	0.252
Milk and milk products (g or ml)	269.5 (217.7–342.2)	277.0 (227.7–349.9)	236.8 (183.0–318.8)	0.070
Fats and oils (g)	29.9 (22.5–35.6)	30.7 (25.4–36.8)	21.1 (16.3–29.0)	0.001
Sugar (g)	8.2 (5.0–11.7)	8.9 (5.0–12.3)	7.1 (4.8–10.0)	0.265

*Nutrients*				
Energy (kcal)	1902 (1625–2131)	1977 (1749–2155)	1484 (1317–1762)	<0.001
Protein (g)	52.2 (43.7–57.1)	54.1 (48.6–59.5)	41.6 (34.8–49.3)	<0.001
% of energy intake	10.9	10.9	10.9	
Fat (g)	58.3 (50.6–66.0)	60.0 (53.4–70.5)	46.1 (35.2–58.6)	<0.001
% of energy intake	28.1	28.4	27.2	
Carbohydrates (g)	263.3 (232.0–299.7)	278.1 (238.1–310.0)	218.0 (197.7–250.0)	<0.001
% of energy intake	56.4	56.0	57.8	
Fiber (g)	28.9 (24.1–33.3)	30.3 (25.4–34.2)	21.0 (17.2–28.8)	<0.001
Vitamin A (*µ*g)	424.1 (290.1–646.0)	476.7 (323.2–680.4)	324.3 (210.7–439.0)	0.012
Thiamine (mg)	1.03 (0.87–1.19)	1.07 (0.92–1.2)	0.8 (0.66–1.0)	<0.001
Riboflavin (mg)	0.86 (0.71–1.02)	0.9 (0.75–1.0)	0.72 (0.63–0.83)	0.005
Niacin (mg)	8.4 (7.0–9.8)	8.8 (7.3–10.2)	6.6 (5.7–7.9)	<0.001
Vitamin B6 (mg)	1.0 (0.85–1.2)	1.08 (0.9–1.24)	0.82 (0.68–0.92)	<0.001
Folate (*µ*g)	227.4 (186.3–274.6)	237.6 (198.2–280.7)	179.5 (146.5–226.3)	0.001
Vitamin B12 (*µ*g)	0.54 (0.41–1.01)	0.55 (0.41–1.03)	0.5 (0.43–0.96)	0.984
Vitamin C (mg)	73.6 (55.2–92.0)	81.8 (57.6–96.3)	57.3 (37.5–74.6)	0.003
Calcium (mg)	590.3 (473.5–706.6)	623.7 (522.0–729.5)	482.8 (394.7–593.8)	0.002
Phosphorus (mg)	932.3 (776.4–1066.3)	969.6 (848.3–1079.7)	742.6 (613.2–875.3)	<0.001
Iron (mg)	11.0 (8.7–12.6)	11.3 (9.8–13.1)	8.4 (5.9–11.1)	<0.001
Zinc (mg)	6.8 (5.9–8.1)	7.2 (6.2–8.2)	5.4 (4.7–6.7)	<0.001
Sodium (mg)	315.9 (268.5–381.1)	329.0 (290.9–389.7)	262.4 (217.1–294.3)	<0.001
Potassium (mg)	1825.9 (1538.2–2222.7)	1899.5 (1619.3–2325.4)	1384.7 (1088.6–1647.5)	<0.001
Selenium (*µ*g)	36.0 (25.8–48.6)	38.9 (29.5–49.0)	24.4 (17.9–41.0)	0.013

*P*_25_: 25th percentile; *P*_75_: 75th percentile. Values represent medians, 25th and 75th percentiles, and are expressed per day. *P* < 0.05 was considered to be significant.

## Data Availability

No data were used to support the findings of this study.

## Abbreviations

HGS: Handgrip strength
GS: Gait speed
BMI: Body mass index
FBG: Fasting blood glucose
UACR: Urinary albumin-to-creatinine ratio
PA: Probability of adequacy
MPA: Mean probability of adequacy
EAR: Estimated average requirement.

## Supplementary Materials

Supplementary Materials Supplementary Table 1A: cutoff values for grip strength for diagnosing frailty. Supplementary Table 1B: cutoff values for gait speed for diagnosing frailty. Supplementary Table 2: quality of food and nutrient intake of the participants in the study. Supplementary Table 3A: association of intake of food groups with frailty status of the participants. Supplementary Table 3B: association of intake of nutrients with frailty status of the participants.
