# Supplementary material for: Frailty and Nutritional Status among Urban Older Adults in South India
Source: J Aging Res. 2020 Jul 10;2020:8763413. doi: 10.1155/2020/8763413 (PMC7368949; doi:10.1155/2020/8763413)
Supplement: Supplementary Materials — Supplementary Table 1A: cutoff values for grip strength for diagnosing frailty. Supplementary Table 1B: cutoff values for gait speed for diagnosing frailty. Supplementary Table 2: quality of food and nutrient intake of the participants in the study. Supplementary Table 3A: association of intake of food groups with frailty status of the participants. Supplementary Table 3B: association of intake of nutrients with frailty status of the participants. [file 8763413.f1.docx]

**Supplementary Material**

**Frailty and nutritional status among urban older adults in South India**

T. Shalini^1^, P. Swathi Chitra^1^, B. Naveen Kumar^2^, G. Madhavi^3^, G. Bhanuprakash Reddy^1#^

Departments of ^1^Biochemistry, ^2^Statistics, ^3^Community Studies, ICMR-National Institute of Nutrition, Jamai-Osmania, Tarnaka, Hyderabad, Telangana, India.

**Supplementary Table 1A:** Cut-off values for grip strength for diagnosing frailty

| BMI | Grip strength (Kg) criterion for frailty |
| --- | --- |
| Men |  |
| BMI, 18.5-22.99 kg/m^2^ | ≤6 |
| BMI, ≥23.0 kg/m^2^ | ≤13 |
| Women |  |
| BMI, <18.5 kg/m^2^ | ≤1 |
| BMI, 18.5-22.99 kg/m^2^ | ≤1 |
| BMI, ≥23.0 kg/m^2^ | ≤2 |

BMI: body mass index

**Supplementary Table 1B:** Cut-off values for gait speed for diagnosing frailty

|  | Seconds taken to walk ten-meter criterion for frailty |
| --- | --- |
| Men |  |
| Height, <165 cm | ≥12 |
| Height, ≥165 cm | ≥11 |
| Women |  |
| Height, <152 cm | ≥15 |
| Height, ≥152 cm | ≥15 |

**Supplementary Table 2:** Quality of food and nutrient intake of the participants in the study.

| Nutrients | Density (per 1000 Kcal) | | p-value | Per kg body weight | | p-value |
| --- | --- | --- | --- | --- | --- | --- |
|  | Non-frail (n=68)  Median (P_25_-P_75_) | Frail (n=20)  Median (P_25_-P_75_) |  | Non-frail (n=68)  Median (P_25_-P_75_) | Frail (n=20)  Median (P_25_-P_75_) |  |
| Energy (Kcal) | - | - | - | 28.2 (24.9-33.7) | 28.3 (19.7-34.4) | 0.544 |
| Protein (g) | 26.8 (25.8-28.1) | 26.9 (25.6-28.1) | 0.850 | 0.77 (0.69-0.89) | 0.72 (0.56-0.92) | 0.370 |
| Fat (g) | 31.6 (28.2-34.7) | 30.7(27.3-32.7) | 0.305 | 0.9 (0.78-1.0) | 0.91 (0.54-1.13) | 0.449 |
| Carbohydrate (g) | 139.3 (132.8-147.7) | 144.6 (133.7-151.3) | 0.130 | 3.9 (3.5-4.8) | 4.2 (3.1-4.9) | 0.811 |
| Fibre (g) | 15.0 (13.8-16.6) | 13.9 (12.8-15.9) | 0.179 | 0.42 (0.37-0.5) | 0.4 (0.28-0.51) | 0.278 |
| Vitamin A (µg) | 251.6 (158.7-353.9) | 203.2 (145.0-273.6) | 0.305 | 6.9 (4.3-10.4) | 5.5 (3.3-8.8) | 0.240 |
| Thiamine (mg) | 0.54 (0.50-0.57) | 0.54 (0.46-0.62) | 0.921 | 0.02 (0.01-0.02) | 0.01 (0.01-0.02) | 0.344 |
| Riboflavin (mg) | 0.43 (0.4-0.49) | 0.48 (0.42-0.55) | 0.116 | 0.01 (0.01-0.01) | 0.01 (0.01-0.02) | 0.498 |
| Niacin (mg) | 4.4 (4.2-4.8) | 4.3 (4.2-4.6) | 0.598 | 0.13 (0.11-0.15) | 0.12 (0.09-0.16) | 0.505 |
| Vitamin B6 (mg) | 0.54 (0.47-0.61) | 0.53 (0.48-0.58) | 0.647 | 0.02 (0.01-0.02) | 0.01 (0.01-0.02) | 0.329 |
| Folate (µg) | 103.1 (89.2-122.8) | 96.3 (87.5-107.3) | 0.160 | 2.9 (2.5-3.7) | 2.7 (1.8-3.8) | 0.135 |
| Vitamin B12 (µg) | 0.27 (0.22-0.49) | 0.35 (0.26-0.58) | 0.072 | 0.01 (0.01-0.02) | 0.01 (0.01-0.02) | 0.232 |
| Vitamin C (mg) | 39.1 (32.0-49.8) | 39.1 (26.-44.3) | 0.409 | 1.12 (0.84-1.5) | 1.18 (0.62-1.44) | 0.386 |
| Calcium (mg) | 309.0 (268.8-352.9) | 322.8 (272.4-377.9) | 0.647 | 8.9 (7.2-10.9) | 9.5 (6.6-10.9) | 0.873 |
| Phosphorus (mg) | 493.2 (457.4-518.4) | 482.3 (466.0-534.9) | 0.960 | 13.6 (12.6-15.8) | 13.2 (10.1-16.0) | 0.305 |
| Iron (mg) | 5.6 (5.2-6.4) | 5.3 (4.3-6.2) | 0.094 | 0.16 (0.14-0.19) | 0.14 (0.1-0.22) | 0.109 |
| Zinc (mg) | 3.7 (3.5-3.8) | 3.5 (3.3-3.8) | 0.125 | 0.1 (0.09-0.12) | 0.1 (0.07-0.12) | 0.265 |
| Sodium (mg) | 175.3 (155.7-194.2) | 180.2 (134.8-197.1) | 0.505 | 4.8 (4.3-6.0) | 4.4 (3.2-6.2) | 0.248 |
| Potassium (mg) | 964.5 (874-1098.7) | 902.5 (781.5-988.2) | 0.023 | 27.1 (23.3-34.1) | 24.3 (18.0-30.4) | 0.057 |
| Selenium (µg) | 20.7 (14.6-25.3) | 15.8 (12.7-23.0) | 0.202 | 0.6 (0.42-0.73) | 0.42 (0.27-0.79) | 0.192 |

**Supplementary Table 3A.** Association of intake of food groups with frailty status of the participants

| Food groups | Unweighted (%) | p-value | Model 1 (%) |  | p-value |
| --- | --- | --- | --- | --- | --- |
| Cereals & millets (g) |  |  |  |  |  |
| Tertile 1 (T_1_) (<208.67) | 41.4 | 0.006 | 46.4 |  | 0.004 |
| Tertile 2 (T_2_) (208.67-267.43) | 20.7 |  | 25.9 |  |  |
| Tertile 3 (T_3_) (>267.43) | 6.7 |  | 5.8 |  |  |
| Pulses & legumes (g) |  |  |  |  |  |
| T_1_ (<30.83) | 48.3 | <0.001 | 54.2 |  | <0.001 |
| T_2_ (30.83-45.60) | 13.8 |  | 18.7 |  |  |
| T_3_ (>45.60) | 6.7 |  | 7.4 |  |  |
| Green leafy vegetables (g) |  |  |  |  |  |
| T_1_ (<13.51) | 41.4 | 0.014 | 52.3 |  | <0.001 |
| T_2_ (13.51-27.63) | 13.8 |  | 14.9 |  |  |
| T_3_ (>27.63) | 13.3 |  | 18.5 |  |  |
| Other vegetables (g) |  |  |  |  |  |
| T_1_ (<95.92) | 24.1 | 0.764 | 32.7 |  | 0.057 |
| T_2_ (95.92-152.12) | 24.1 |  | 35.3 |  |  |
| T_3_ (>152.12) | 17.2 |  | 18.3 |  |  |
| Roots & tubers (g) |  |  |  |  |  |
| T_1_ (<46.79) | 34.5 | 0.145 | 42.0 |  | 0.131 |
| T_2_ (46.79-72.24) | 20.7 |  | 25.3 |  |  |
| T_3_ (>72.24) | 13.3 |  | 19.4 |  |  |
| Nuts & oilseeds (g) |  |  |  |  |  |
| T_1_ (<5.40) | 37.9 | 0.016 | 43.7 |  | 0.007 |
| T_2_ (5.40-11.2) | 24.1 |  | 32.7 |  |  |
| T_3_ (>11.2) | 6.7 |  | 8.2 |  |  |
| Spices & condiments (g) |  |  |  |  |  |
| T_1_ (<9.35) | 44.8 | 0.001 | 51.2 |  | 0.006 |
| T_2_ (9.35-12.2) | 20.0 |  | 22.2 |  |  |
| T_3_ (>12.2) | 3.4 |  | 6.8 |  |  |
| Fruits (g) |  |  |  |  |  |
| T_1_ (<99.43) | 31.0 | 0.117 | 41.8 |  | 0.018 |
| T_2_ (99.43-162.33) | 27.6 |  | 33.9 |  |  |
| T_3_ (>162.33) | 10.0 |  | 10.9 |  |  |
| Animal foods (g) |  |  |  |  |  |
| T_1_ (<0.0) | 25.6 | 0.620 | 34.9 |  | 0.622 |
| T_2_ (0.0-35.6) | 26.3 |  | 28.9 |  |  |
| T_3_ (>35.6) | 16.7 |  | 22.2 |  |  |
| Milk & milk products (g or mL) |  |  |  |  |  |
| T_1_ (<233.12) | 34.5 | 0.155 | 38.9 |  | 0.199 |
| T_2_ (233.12-315.36) | 13.8 |  | 18.7 |  |  |
| T_3_ (>315.36) | 20.0 |  | 28.4 |  |  |
| Fats & oils (g) |  |  |  |  |  |
| T_1_ (<25.39) | 41.4 | 0.011 | 53.4 |  | <0.001 |
| T_2_ (25.39-32.84) | 17.2 |  | 19.1 |  |  |
| T_3_ (>32.84) | 10.0 |  | 10.6 |  |  |
| Sugars (g) |  |  |  |  |  |
| T_1_ (<5.24) | 25.9 | 0.119 | 31.4 |  | 0.123 |
| T_2_ (5.24-10.0) | 34.5 |  | 44.5 |  |  |
| T_3_ (>10.0) | 11.1 |  | 17.2 |  |  |

T_1_: Tertile 1; T_2_: Tertile 2; T_3_: Tertile 3

Model 1: adjusted for age and gender

Values represent percentages (%). p<0.05 was considered to be significant.

**Supplementary Table 3B.** Association of intake of nutrients with frailty status of the participants.

| Nutrients | Unweighted (%) | p-value | Model 1 (%) | p-value | Model 2 (%) | p-value |
| --- | --- | --- | --- | --- | --- | --- |
| Energy (Kcal) |  |  |  |  |  |  |
| T_1_ (<1718) | 48.3 | <0.001 | 52.4 | <0.001 |  | - |
| T_2_ (1718-2031) | 17.2 |  | 19.2 |  | - |  |
| T_3_ (>2031) | 3.3 |  | 3.5 |  |  |  |
| Protein (g) |  |  |  |  |  |  |
| T_1_ (<46.16) | 51.7 | <0.001 | 57.3 | <0.001 | 55.2 | <0.001 |
| T_2_ (46.16-55.72) | 13.8 |  | 13.8 |  | 14.0 |  |
| T_3_ (>55.72) | 3.3 |  | 3.0 |  | 2.7 |  |
| Fat (g) |  |  |  |  |  |  |
| T_1_ (<53.18) | 48.3 | <0.001 | 54.7 | <0.001 | 51.1 | <0.001 |
| T_2_ (53.18-64.61) | 10.3 |  | 12.4 |  | 11.9 |  |
| T_3_ (>64.61) | 10.0 |  | 10.9 |  | 10.1 |  |
| Carbohydrates (g) |  |  |  |  |  |  |
| T_1_ (<237.82) | 44.8 | <0.001 | 50.4 | <0.001 | 47.0 | <0.001 |
| T_2_ (237.82-281.6) | 24.1 |  | 26.6 |  | 26.3 |  |
| T_3_ (>281.6) | 0.0 |  | 0.0 |  | 0.0 |  |
| Fibre (g) |  |  |  |  |  |  |
| T_1_ (<24.74) | 48.3 | <0.001 | 55.3 | <0.001 | 51.3 | 0.001 |
| T_2_ (24.74-31.16) | 10.3 |  | 10.2 |  | 10.2 |  |
| T_3_ (>31.16) | 10.0 |  | 11.3 |  | 10.0 |  |
| Vitamin A (µg) |  |  |  |  |  |  |
| T_1_ (<338.34) | 34.5 | 0.079 | 43.9 | <0.001 | 35.9 | 0.039 |
| T_2_ (338.34-551.1) | 24.1 |  | 31.7 |  | 28.2 |  |
| T_3_ (>551.1) | 10.0 |  | 11.2 |  | 10.2 |  |
| Thiamine (mg) |  |  |  |  |  |  |
| T_1_ (<0.9) | 46.4 | 0.001 | 50.5 | <0.001 | 47.4 | 0.001 |
| T_2_ (0.9-1.13) | 12.5 |  | 14.8 |  | 14.0 |  |
| T_3_ (>1.13) | 10.7 |  | 11.7 |  | 10.4 |  |
| Riboflavin (mg) |  |  |  |  |  |  |
| T_1_ (<0.74) | 44.8 | 0.002 | 47.8 | 0.001 | 44.1 | 0.001 |
| T_2_ (0.74-0.92) | 10.7 |  | 18.3 |  | 14.6 |  |
| T_3_ (>0.92) | 12.9 |  | 15.3 |  | 13.7 |  |
| Niacin (mg) |  |  |  |  |  |  |
| T_1_ (<7.27) | 44.8 | 0.002 | 50.9 | <0.001 | 47.9 | 0.001 |
| T_2_ (7.27-9.22) | 16.7 |  | 17.0 |  | 16.3 |  |
| T_3_ (>9.22) | 6.9 |  | 8.6 |  | 7.4 |  |
| Vitamin B6 (mg) |  |  |  |  |  |  |
| T_1_ (<0.89) | 48.3 | <0.001 | 55.4 | <0.001 | 50.5 | <0.001 |
| T_2_ (0.89-1.14) | 14.3 |  | 13.1 |  | 13.2 |  |
| T_3_ (>1.14) | 6.5 |  | 11.4 |  | 9.0 |  |
| Folate (µg) |  |  |  |  |  |  |
| T_1_ (<197.88) | 33.3 | 0.032 | 40.9 | <0.001 | 36.7 | <0.001 |
| T_2_ (197.88-258.87) | 11.5 |  | 16.0 |  | 13.3 |  |
| T_3_ (>258.87) | 7.1 |  | 5.7 |  | 5.6 |  |
| Vitamin B12 (µg) |  |  |  |  |  |  |
| T_1_ (<0.45) | 20.7 | 0.948 | 25.1 | 0.724 | 21.7 | 0.786 |
| T_2_ (0.45-0.85) | 24.1 |  | 35.1 |  | 28.5 |  |
| T_3_ (>0.85) | 23.3 |  | 30.2 |  | 25.0 |  |
| Vitamin C (mg) |  |  |  |  |  |  |
| T_1_ (<59.26) | 37.9 | 0.016 | 46.5 | 0.003 | 39.2 | 0.036 |
| T_2_ (59.26-86.53) | 24.1 |  | 31.7 |  | 28.7 |  |
| T_3_ (>86.53) | 6.7 |  | 7.8 |  | 6.8 |  |
| Calcium (mg) |  |  |  |  |  |  |
| T_1_ (<518.79) | 41.4 | 0.006 | 45.3 | 0.004 | 39.7 | 0.007 |
| T_2_ (518.79-699.14) | 20.7 |  | 26.9 |  | 25.0 |  |
| T_3_ (>699.14) | 6.7 |  | 8.6 |  | 7.7 |  |
| Phosphorus (mg) |  |  |  |  |  |  |
| T_1_ (<845.82) | 44.8 | 0.002 | 48.5 | <0.001 | 44.7 | 0.001 |
| T_2_ (845.82-1000.1) | 17.2 |  | 20.4 |  | 20.0 |  |
| T_3_ (>1000.1) | 6.7 |  | 6.0 |  | 5.4 |  |
| Iron (mg) |  |  |  |  |  |  |
| T_1_ (<9.45) | 48.3 | <0.001 | 54.6 | <0.001 | 50.9 | 0.001 |
| T_2_ (9.45-11.85) | 6.9 |  | 5.4 |  | 5.3 |  |
| T_3_ (>11.85) | 13.3 |  | 16.2 |  | 14.7 |  |
| Zinc (mg) |  |  |  |  |  |  |
| T_1_ (<6.13) | 48.3 | <0.001 | 53.3 | <0.001 | 50.6 | <0.001 |
| T_2_ (6.13-7.82) | 13.8 |  | 14.1 |  | 13.9 |  |
| T_3_ (>7.82) | 6.7 |  | 6.2 |  | 5.6 |  |
| Sodium (mg) |  |  |  |  |  |  |
| T_1_ (<283.43) | 44.8 | 0.002 | 48.7 | <0.001 | 44.9 | <0.001 |
| T_2_ (283.43-349.22) | 13.8 |  | 17.4 |  | 15.1 |  |
| T_3_ (>349.22) | 10.0 |  | 13.1 |  | 11.7 |  |
| Potassium (mg) |  |  |  |  |  |  |
| T_1_ (<1588.33) | 48.3 | <0.001 | 53.0 | <0.001 | 49.6 | <0.001 |
| T_2_ (1588.3-1994.68) | 17.2 |  | 21.2 |  | 19.5 |  |
| T_3_ (1994.68) | 3.3 |  | 2.7 |  | 2.6 |  |
| Selenium (µg) |  |  |  |  |  |  |
| T_1_ (<29.13) | 44.8 | 0.002 | 55.4 | <0.001 | 50.5 | 0.011 |
| T_2_ (29.13-45.38) | 6.9 |  | 9.5 |  | 7.9 |  |
| T_3_ (>45.38) | 16.7 |  | 17.6 |  | 15.3 |  |

T_1_: Tertile 1; T_2_: Tertile 2; T_3_: Tertile 3

Model 1: adjusted for age and gender. Model 2: adjusted for age, gender and energy

Values represent percentages (%). p<0.05 was considered to be significant.
